# Woodhall Spa

**Published:** 1887-04-23

**Authors:** 


### WOODHALL SPA.

Dr. C. T. Williams, the Medical Superintendent at the Hydropathic Establishment, Woodhall Spa, Horncastle, writes:—"Seeing in your issue of February 12th certain questions relating to the curative and medicinal properties of the Woodhall Spa water, I have much pleasure in informing 'Enquirer' that this water is specially recommended by the medical profession for the following complaints:—Rheumatism, gout, scrofula, chronic skin diseases, tumours, and diseases peculiar to women, whilst the large amount of iodine contained in it has been found to render it of the greatest service in glandular enlargements. Great efforts are being made to bring before the public the extraordinary virtues of the Spa, by the erection of new baths, with all the most recent appliances, and by circulating more largely this water, which has so long remained comparatively unknown. An analysis made in November last by Professor Wanklyn shows that the water is richer in bromides and iodides than any other in Europe, whilst his discovery of the interesting fact that iodine exists in its free state, is a subject of great therapeutical value. I may add that a hospital supported by voluntary contributions is to be erected at Woodhall for the accommodation of poorer patients."

Mrs. Hotchkin writes from The Priory, Dursley, as follows:—"Having seen your query in The Hospital with reference to Woodhall Spa, which is well known to me, I would refer you for the fullest information possible, to the Rev John O. Stephens, M.A., Rector of Blankney. The Woodhall waters are the strongest known bromo-iodine mineral waters in Europe. They are especially recommended by the Faculty for the cure of scrofula, pulmonary consumption in its early stages, of rheumatism in all its forms, rheumatic gout, gout, tumours, glandular swellings, epilepsy, skin-diseases, indigestion, etc."

